# Inference of the genetic basis of fruit texture in highbush blueberries using genome-wide association analyses

**DOI:** 10.1093/hr/uhae233

**Published:** 2024-08-21

**Authors:** Luis Felipe V Ferrão, Camila Azevedo, Juliana Benevenuto, Molla Fentie Mengist, Claire Luby, Marti Pottorff, Gonzalo I P Casorzo, Ted Mackey, Mary Ann Lila, Lara Giongo, Nahla Bassil, Penelope Perkins-Veazie, Massimo Iorizzo, Patricio R Munoz

**Affiliations:** Blueberry Breeding and Genomics Lab, Horticultural Sciences Department, University of Florida, Gainesville, FL 32611, USA; Blueberry Breeding and Genomics Lab, Horticultural Sciences Department, University of Florida, Gainesville, FL 32611, USA; Statistic Department, Federal University of Vicosa, Vicosa, Brazil; Blueberry Breeding and Genomics Lab, Horticultural Sciences Department, University of Florida, Gainesville, FL 32611, USA; Blueberry Breeding and Genomics Lab, Horticultural Sciences Department, University of Florida, Gainesville, FL 32611, USA; Plants for Human Health Institute, North Carolina State University, Kannapolis, NC USA; USDA-ARS, Horticulture Crops Research Unit, Corvallis, OR 97333, USA; Blueberry Breeding and Genomics Lab, Horticultural Sciences Department, University of Florida, Gainesville, FL 32611, USA; USDA-ARS, Horticulture Crops Research Unit, Corvallis, OR 97333, USA; Plants for Human Health Institute, North Carolina State University, Kannapolis, NC USA; Fondazione Edmund Mach - Research and Innovation Centre Italy; USDA-ARS, Horticulture Crops Research Unit, Corvallis, OR 97333, USA; Plants for Human Health Institute, North Carolina State University, Kannapolis, NC USA; Plants for Human Health Institute, North Carolina State University, Kannapolis, NC USA; Blueberry Breeding and Genomics Lab, Horticultural Sciences Department, University of Florida, Gainesville, FL 32611, USA

## Abstract

The global production and consumption of blueberry (*Vaccinium* spp.), a specialty crop known for its abundant bioactive and antioxidant compounds, has more than doubled over the last decade. To hold this momentum, plant breeders have begun to use quantitative genetics and molecular breeding to guide their decisions and select new cultivars that are improved for fruit quality. In this study, we leveraged our inferences on the genetic basis of fruit texture and chemical components by surveying large breeding populations from northern highbush blueberries (NHBs) and southern highbush blueberries (SHBs), the two dominant cultivated blueberries. After evaluating 1065 NHB genotypes planted at the Oregon State University, and 992 SHB genotypes maintained at the University of Florida for 17 texture-related traits, evaluated over multiple years, our contributions consist of the following: (i) we drew attention to differences between NHB and SHB materials and showed that both blueberry types can be differentiated using texture traits; (ii) we computed genetic parameters and shed light on the genetic architecture of important texture attributes, indicating that most traits had a complex nature with low to moderate heritability; (iii) using molecular breeding, we emphasized that prediction could be performed across populations; and finally (iv) the genomic association analyses pinpointed some genomic regions harboring potential candidate genes for texture that could be used for further validation studies. Altogether, the methods and approaches used here can guide future breeding efforts focused on maximizing texture improvements in blueberries.

## Introduction

Blueberry (*Vaccinium corymbosum* and hybrids) is a crop with expanding production and consumption worldwide. Considered a rich source of bioactive and antioxidant compounds, blueberry’s status as a “superfood” has been a major factor driving the increase of production and consumption [1]. In the USA, for example, consumption of blueberries has experienced a more than a 300% increase in per capita consumption since 2005 and is now at an all-time high of over 0.9 kg per person. Globally, blueberry production has more than doubled over the last decade.

Several species are commercially important in the *Vaccinium* genus. The *V. corymbosum* L. is the dominant cultivated species and can be further separated into northern highbush blueberries (NHBs) and southern highbush blueberries (SHBs), depending on their chilling requirements and winter hardiness. NHB is native to the Eastern and Mid-Western regions of the USA and requires more than 800 hours of chilling at 0–7°C to break bud dormancy. Commercially, NHB cultivars are grown across the Northern regions of the USA, as well as in Canada, Chile, New Zealand, Northern Europe, and China. Most of the current cultivars of NHB were developed in New Jersey, North Carolina, and Michigan; however, as production has been increasing in the Pacific Northwest, there are now several public and private breeding programs there. Starting in 1950, the breeding program based at the University of Florida led pioneering hybridizations between NHB and species native and adapted to the southeastern USA (e.g. *Vaccinium darrowii*) aiming for better adaptation to higher temperatures and disease pressure [2]. This introgression led to a reduction in the chilling hours required to break flower bud and ultimately established a new blueberry varietal group, the southern highbush (SHB). To date, new plantings of SHB in low chilling geographic regions has been the main driver of the global expansion of blueberry production. SHB plants establish more quickly than NHB, thus producing fruit earlier. Thus, precociousness together with the early-ripening cultivars enable growers to reach an early market window, when prices are higher and competition is lower, making the crop more profitable for growers in tropical and subtropical regions of Africa, America, and Asia [3].

To maintain the marketability of both NHB and SHB cultivars, the blueberry industry seeks to meet the current demand for improved fruit quality [4]. Good texture is considered a key element, affecting both consumer appeal and postharvest decay. Despite the importance of this trait, the genetic improvements for texture have experienced slow progress in the past few decades. This is primarily due to the difficulty in phenotyping the “texture trait.” For example, trained panelists can define texture descriptors and associate them to sensorial perception, but such work is costly and difficult to scale to large breeding populations [5]. As an alternative, instrumental tools have been used to measure texture, leveraging the higher throughput data collection and working as a proxy of sensory evaluations. In blueberries, the most common method used to evaluate texture is the FirmTech instrument which measure a single parameter commonly called “Firmness” (slope between two predefined points on the force-deformation curve) and assume that texture in blueberry is defined by a single component [6]. Although able to differentiate firmness categories (i.e., firm and soft fruits), such equipment is not able to dissect multiple texture components.

**Figure 1:**
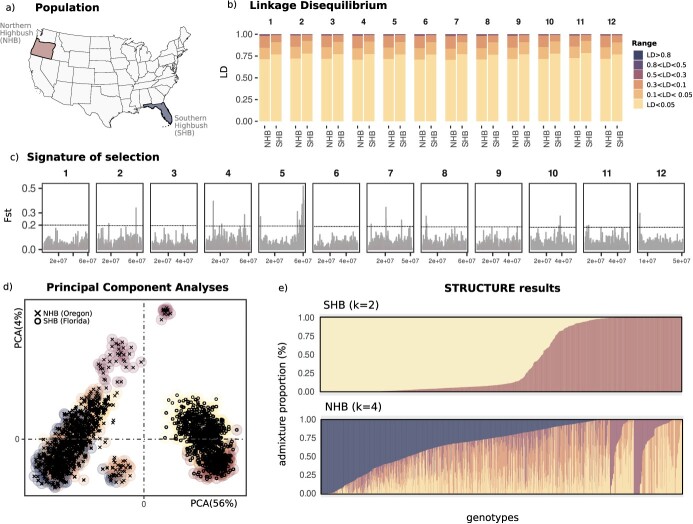
(a) The two cultivated blueberry populations, used in this study: Southern Highbush (SHB) evaluated in Florida, and Northen Highbush (NHB) evaluated in Oregon. (b) LD (r2) values frequency computed by chromosome across both populations. (c) Genome-wide signature of selection using the Fst statistics, the horizontal line shows a conservative threshold of 0.2. (d) Genetic diversity within the blueberry germplasm using PCA; (e) Population structure evaluated in each blueberry population using an admixture model. Individuals in the subpopulations represented in the PCA plot were colored based on the population structure results.

**Table 1:** Texture, parameters evaluated using the texture analyzer (TA) instruments in bluberries

**Method**	**Texture parameter**	**Abbreviation**	**Description**	**Unit**
Penetration with 2 mm flat probe	Force at 1 mm	F1mm	Force at 1 mm depth	N
	Maximum force	FM	Force at point of skin puncture	N
	Distance to maximum force	DFM	Distance between point of contact and skin puncture	mm
	Slope maximum force	SFM	Slope between force at 1 mm and maximum force	N mm^−1^
	Area maximum force	AFM	Area underlying the curve between point of contact and maximum force	N mm
	Linear distance to maximum force	LDFM	Length of curve between point of contact and maximum force	-
	Mean internal force	MIF	Mean force between first peak after minimum force* and 80% strain	N
	Area internal force	AIF	Area underlying the curve between first peak after minimum force and 80% strain	N mm
	Number of internal peaks	NIP	Number of peaks between first peak after minimum force and 80% strain (threshold: 1 g)	-
	Force linear distance	FLD	Length of curve between point of contact and 80% strain	-
	Area force linear distance	AFLD	Area between point of contact and 80% strain	N mm
	Burst strain	BrSt	Strain at maximum force	%
	Young’s modulus 10%	YM10PD_Cond	Slope between force at minimum time and force at 10% strain**	MPa/%
	Young’s modulus 20% burst strain	YM20_BrSt	Slope between force at minimum time and force at 20% of BrSt	MPa/%
	Young’s modulus 80% burst strain	YM80_BrSt	Slope between force at minimum time and force at 80% of BrSt	MPa/%
	Young’s modulus 100% burst strain	YM100_BrSt	Slope between force at minimum time and force at 100% of BrSt	MPa/%
	Young’s modulus 1–2%	YM1to2	Slope between force at 1% and 2% strain	MPa/%

More recently, texture analyzer (TA) instruments have been proposed as a better tool for the description of texture profiles in fruits and vegetables. TA instruments have the potential to simultaneously measure multiple mechanical texture components and acoustic attributes and therefore they can distinguish genotypes with singular texture profiles in blueberries. Using TA, multiple studies have demonstrated that multiple components define blueberry texture [7, 8] which highlighted the added value of using this method. When associated to genomic information further benefits can be projected in a practical breeding program. Breeders could use molecular markers to predict texture attributes, which can maximize the genetic gains by leveraging the selective accuracy and reducing the breeding cycles. So far, the use of genomic-assisted breeding has been proposed for different fruit quality traits in blueberry but not for more specific texture attributes [9, 10].

In this study, we hypothesize that (i) genetic variants associated to different mechanical texture parameters measured by the TA could be identified via genome-wide association study (GWAS) to understand their genetic architecture; and that (ii) blueberry genotypes with outstanding texture attributes could be predicted using molecular breeding to enhance genetic gain. To test it, we combined a target genotyping approach and TA measurements from a total of 2289 individuals, comprising NHB and SHB breeding populations, evaluated over 2 years. Our contributions in this article are 3-fold: (i) we estimated genetic parameters and provided new insights on the genetic architecture of texture traits; (ii) using GWAS, we pointed out genomic regions controlling texture attributes and plausible candidate genes; and finally (iii) given the difficulty of phenotyping texture, we demonstrated the potential of genomic prediction to be implemented at the scope of NHB and SHB breeding programs. To our knowledge, this is the first and largest study assessing the genetic architecture of different mechanical texture parameters in blueberry.

## Results

### Population structure and genetic diversity in cultivated blueberries

To elucidate the genetic basis of texture traits, we investigated two large blueberry populations separated into NHB and SHB types (Fig. 1a). This is the first time in the fruit literature that a such large and representative population was considered for genetic analyses of texture attributes. Therefore, we first focused on estimating some population genetic parameters. The linkage disequilibrium (LD) was computed and represented in the form of frequency by chromosome (Fig. 1b). Overall, in both groups, less than 1% of pairwise markers showed higher LD (>0.8), and a fast LD decay was observed across all the chromosomes. This LD pattern is expected since both populations are highly diverse, containing individuals in early evaluation stages in the breeding pipeline. The cross-pollination form, associated to the high inbreeding depression observed in the species, are also two important sources that leverage the genetic diversity and affect the LD results. We also investigated potential genomic regions that are differentially selected in both populations. After computing genome wide Fst between both blueberry groups, several regions across the chromosomes showed signatures of selection (Fig. 1c). The selective sweep observed at the end of the chromosome 5 had the strongest peak.

To explore the relationship between individuals within and across populations, we first evaluated the genetic diversity using principal component analyses (PCAs). The first two dimensions (PC1 and PC2) explained 56% and 4% of the structural variance, respectively. Two distinct genetic groups were identified, with SHB genotypes positioned in the quadrants I and IV; and the NHB in the quadrants II and III. To complement the within-population analyses, we relied on the probabilistic models implemented in the STRUCTURE software (Fig. 1e). For the SHB population, a sub-structured was observed within this genetic pool, in which two subgroups were identified. A large amount of admixture was observed in the NHB collection with four subgroups identified.

### Texture variation in SHB and NHB populations

In this study, we investigated the genetic diversity of 17 texture parameters evaluated in two large breeding populations of NHB and SHB blueberries (Table 1). The NHB population was part of the USDA-ARS and Oregon State University Cooperative breeding program, and the population was divided into a group of 113 individuals, named here as Validation study set (VAL set), which included advanced selections and cultivars, and 952 individuals, named here as Genetic study set (GEN set), consisting of early selections derived from multiple families (Fig. 1a). Similar genetic material was used for the SHB, which were part of the breeding program at the University of Florida. SHB populations included 113 advanced selection and cultivars as part of the VAL set, and 879 early selections as part of the GEN set (Fig. 1a). Texture attributes were evaluated using a penetration method implemented in a TA.XTPlus Texture Analyzer (Stable Micro Systems, Hamilton, MA, USA) as previously described by [11]. A total of 17 texture-related metrics were retrieved from the TA and used in this study.

**Figure 2:**
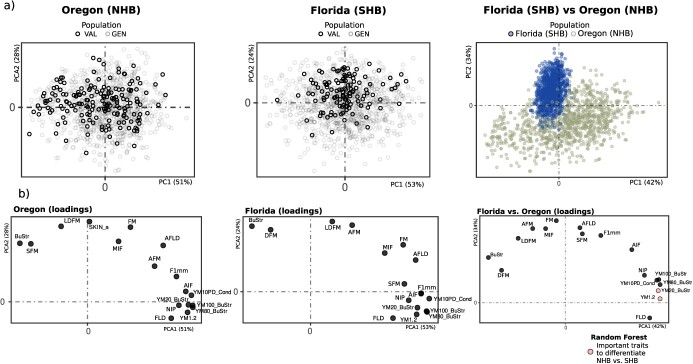
PCA based on 17 texture traits. (a) First two principal components evaluated for populations of Northern Highbush Blueberries (NHB) and Southern Highbush Blueberries (SHB) evaluated in Oregon and Florida, respectively. Each point represents a blueberry genotype, in which the GEN and VAL data sets are represented by different colors. Loading vectors describing how texture traits influence the variation in blueberry data from both locations separately. (b) PCA analyses and loading vectors when both NHB and SHB populations were considered simultaneously. In the loading vectors plot, the highlighted dots represent the most important traits for separating NHB and SHB populations measured via Random Forest model.

In terms of phenotypic variation for texture-related traits, there was considerable diversity for all traits. On average, individuals from the SHB population showed larger values for the difference metrics computed via TA than NHB (Supplemental Material S1), with genotypes in the GEN set, presenting higher phenotypic variation than in VAL set. After combining information from both populations, we assessed the phenotypic diversity of texture attributes using multivariate analyses. In the PCA, we observed a clear distinction between SHB and NHB genotypes (Fig. 2a). Regarding trait importance, the texture attribute related to BuStr and all Young’s modulus (YM) trait-related parameters showed high loading scores in the PCA analysis. We relied on unsupervised analyses to identify the key traits to separate both populations. Using Random Forest, we found that four Young’s modulus trait-related parameters (YM_BuStr and YM1.2) are the most important parameters to separate SHB and NHB genotypes (Fig. 2b).

The relationship between texture-related traits and traditional compression force was examined. For this, we collected 100 berries from multiple genotypes and measured, in the same order, for texture attributes using the TA machine, and compression force (or deflection) using FruitFirm 1000 machine. Among all the TA parameters, SFM showed the highest correlations (0.87) with the compression force (g/mm) by the FruitFirm 1000. The linear equation fitted to transform SFM (N mm^−1^) from TA into compression force (g/mm) from FruitFirm 1000 was: ${y}_{CF}=38.7+248{\beta}_1$, where ${y}_{\mathrm{CF}}$ is the predicted compression force value measured in g/mm and ${\beta}_1$is the SFM value reported via TA.

### Texture traits are predictable and show low-to-moderate heritability values

Blueberry breeders have relied on understanding the genetic architecture of complex traits and the use of genomic prediction to guide parental selection in recurrent selection breeding programs. Genome-wide single-nucleotide polymorphisms (SNPs) were obtained using a targeted genotyping by sequencing approach (Flex-Seq) [12] yielding a total of 113,421 markers used to build a genomic relationship matrix of NHB and SHB populations combined.

**Figure 3:**
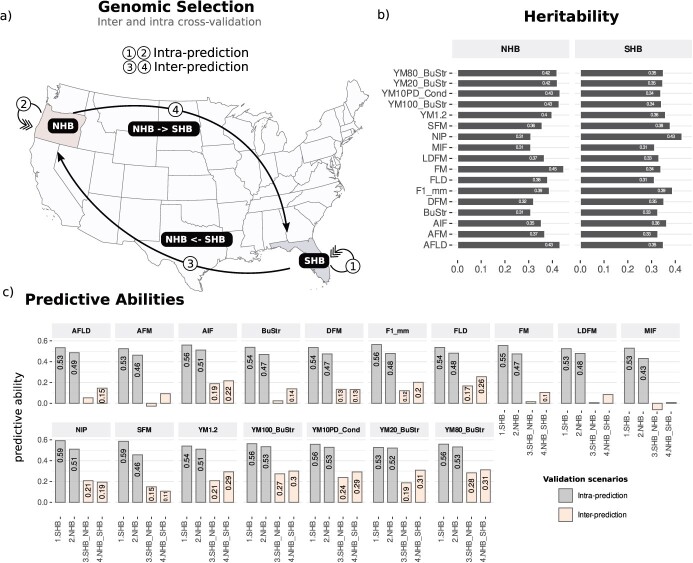
Genomic prediction of texture-related traits in blueberry. (a) Scenarios of cross-validation tested in Northern (NHB) and Southern (SHB) highbush blueberries from Oregon and Florida States, respectively; (b) Genomic heritability for 17 texture traits evaluated in NHB and SHB; (c) Predictive ability measured as the Person’s correlation between predicted and observed phenotypic values for inter and intra-population validation scenarios.

For genomic selection, we first tested four prediction scenarios (Fig. 3a). Firstly, GBLUP models were fitted separately, within NHB and SHB populations (indicated as intra-prediction scenarios)—mimicking the fact the NHB and SHB breeding programs are performed independently. In a more holistic way, models were calibrated in one population and to predict the other in both directions (indicated as inter-prediction scenarios).

The genomic information was also used to estimate heritability values and set up theorical limits for genetic improvements in both populations. Low to moderate genomic heritability values were computed across traits, highlighting the quantitative nature of texture-related traits in blueberries. In general, NHB populations showed higher heritability values than the SHB population (Fig. 3b). Interestingly, some traits presented heterogeneous values across populations, indicating that phenotypic variation in NHB and SHB populations might be under distinct genetic control or that environment has a larger effect on one population than the other. For example, NIP had higher narrow-sense heritability values in SHB when compared to NHB.

Finally, we computed predictive abilities to test the relevance of genomic selection for blueberry texture. When comparing intra-population predictions, SHB showed better prediction abilities for all traits (Fig. 3c). We observed prediction abilities ranging from 0.53 (AFLD, AFM, LDFM, MIF, and YM20_BuStr) to 0.59 (NIP and SFM), with an average value of 0.54. For NHB, values ranged from 0.43 (MIF) to 0.53 (YM100_BuStr, YM10PD_Cond, and YM80_BuStr) with an average value of 0.48. The largest difference between predictive ability was observed for SFM, with an improvement of 0.13 observed in the SHB, when compared to NHB.

Overall, we hypothesized that NHB and SHB breeding programs could be integrated by sharing predictive models across populations. To test it, we compared inter- and intra-population analyses and observed that all prediction abilities were drastically reduced with models calibrated and tested across populations (Fig. 3b). More specifically, for some traits, inter-population will not work regardless of which population the models are calibrated. For example, low predictions were observed for LDFM, AFM, and MIF. For other traits, we could see a directional effect, with models working only when trained in NHB to predict SHB, including AFLD, BuStr, and FM. For all other traits, we could see reasonable results for inter-population predictions, with some traits showing predictive accuracies higher than 0.25 (e.g. YM100_BuStr and YM80_BuStr).

### Texture attributes are consistent across fruits and shows high repeatability values

Another important question is when multiple fruits are evaluated from the same plant, how many samples are necessary to represent a genotype? To answer this question, we estimated the repeatability values based on 10 berries collected from the same genotype, including a permanent environment effect in the mixed model. Like heritability, the repeatability coefficient is also related to the variability in the population, where lower values indicate that a larger number of technical replications (i.e., more berries per plants) must be considered for an efficient selection [13]. All repeatability values computed in both populations were moderate-to-high, indicating that the option for 10 fruits measured during the texture analyses were sufficient to describe the phenotypic variance of a given genotype (Supplemental Fig. S1). Repeatability sets the theoretical upper limits for heritability. In most cases, the heritability values were substantially lower than repeatability estimates, indicating that fruits are relatively uniform within plants, but the environmental variation is an important source of variation in blueberries.

### Genomic regions were found associated with most texture traits

**Figure 4:**
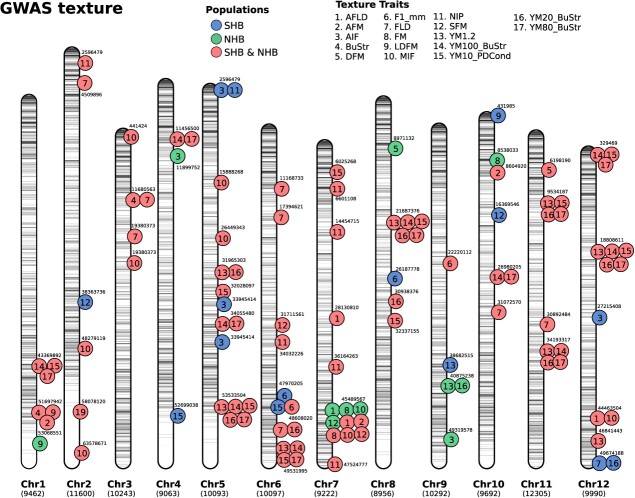
SNP-trait associations detected via GWAS (GWAS). In total, 17 texture- related traits were tested in two blueberry breeding populations (SHB and NHB). GWAS was carried out in the two populations independently and then combining both data sets. Numbers below each chromosome indicate the total number of SNPs. The circles represent the position of a significant marker with the lowest p-value within a window of 5 Mb in the genome. The colors and numbers inside the circles represent the population and traits, respectively, for which the association was detected as indicated in the legend. The numbers outside the circles indicate the position, in base pairs, where the marker with the lowest *P* value was identified.

We performed GWAS analyses for twenty texture-related traits using the Q + K linear mixed and the additive model. Analyses were carried out for each population independently, and then combined SHB and NHB populations (Supplemental Fig. S2, S3, and S4). An illustration displaying the genomic location associated with each trait is summarized in the Fig. 4. The position of the significant markers with the lowest associated *P* value in the genomic region is displayed. In total, we reported 11 genomic regions for NHB, 15 for SHB, and 85 when both SHB and NHB populations were considered together in the GWAS.

Few associations were reported when both populations were evaluated either independently or when both populations were combined for the traits AFM, BuStr, DFM, FM, and LDFM. For all young modulus (YM) texture traits, many significant (>10) associations were reported. The Young modulus parameters (YM1.2, YM10_PDCond, YM20_BuStr, YM80_BuStr, YM100_BuStr) for example showed a high polygenic architecture with hits distributed across 9 different chromosomes. Also, QTL for these parameters clustered around the same regions in chromosome 5 (position 53 533 504), chromosome 6 (position 49 531 955), chromosome 8 (position 21 687 376), chromosome 11 (position 9 534 987 and 34 193 317), and chromosome 12 (position 329 469 and 18 808 611) which suggest similar genetic control. This is consistent with the hypothesis that given that these parameters are representing the same mechanical textural component. Chromosomes 5 and 7 showed the highest number of associations. At the end of the chromosome 7 (position 45 489 567), for example, the same genomic region pinpointed significant associations for 10 different traits. Only for the trait YM1.2, we could map a consistent region across both populations separately, which was located at chromosome 9 with two close genomic regions (position 38 682 515 and 40 875 238).

### Candidate genes were found at significant GWAS regions.

Once significant genomic regions associated with texture-related traits were detected through GWAS analyses, we searched for candidate genes for the most relevant traits, named YM20_BuStr, F1mm, and DFM (Supplementary Table S2). Given the large of number of attributes generated by the TA analyses, we focused our discussion on traits that were identified when both SHB and NHB populations were analyzed together and that have a biological meaning. For example, YM20_BuSt, from the multivariate analyses was revealed as an important trait to differentiate SHB and NHB materials. At the sensory level, F1mm has a good correlation with firmness measured in the consumer panel. Finally, DFM seems an important component for post-harvest.

**Figure 5:**
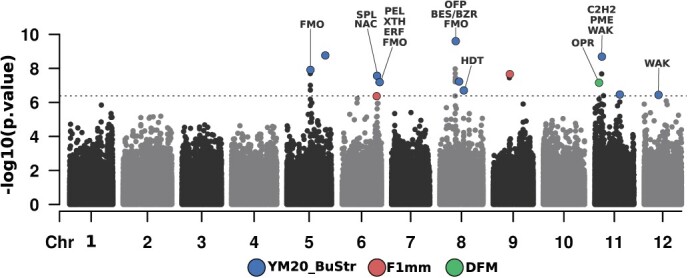
Overlapping Manhattan plots for GWAS hits detected for three texture-related traits (YM20_BuStr, F1mm, and DFM). The dots represent SNPs. The x-axis shows the SNP position across the 12-haploid set of chromosomes of the “W8520_P0” reference genome. The y-axis shows the statistical significance of SNP association with the traits, with dashed line representing threshold at Bonferroni-type correction. Abbreviations of plausible candidate gene names detected in significant GWAS regions are presented as follows: *FMO = flavin-containing monooxygenase; NAC = NAC domain-containing protein; SPL = Squamosa promoter-binding-like protein; ERF = Ethylene-responsive transcription factor; XTH = Xyloglucan endotransglucosylase/hydrolase; PEL = Pectate lyase; BES1/BZR1 = BES1 BZR1 homolog protein; OFP=Ovate family transcription repressor; HDT = histone deacetylase; OPR = 12-oxophytodienoate reductase; WAK = wall-associated receptor kinase; PME = Pectinesterase/pectinesterase inhibitor; C2H2 = C2H2 zinc finger protein*.

Genes directly or indirectly related to cell wall metabolism and fruit firmness variation in other species were identified flanking significant SNPs for YM20_BuStr and DFM traits (Fig. 5). No plausible genes could be pointed for F1mm with the functional description of the genes retrieved so far. The potential roles of these individual genes in texture modification are further elaborated in the discussion section.

## Discussion

In recent decades, numerous breeding schemes have been proposed in plant breeding*.* In general, two main goals are addressed in outcrossing species, such as blueberry: (i) identify the best performing materials for commercial cultivar release and (ii) identify genotypes that can be used as parents in future crosses [9]. To make it more efficient, estimating genetic parameters in diverse populations provides the means to determine the genetic control of complex traits and thus guide future breeding decisions. Although extensive analyses in this area have been reported for SHB [14], and for NHB [15], to our knowledge, no scientific study has addressed populations from both types of blueberry at the same time and considered more detailed texture attributes.

Fruit quality comprises a complex series of traits that contribute to the attractiveness and suitability of fruits for consumption. Texture at maturity is arguably one of the most important attributes for blueberry consumers and retailers, since it contributes to consumer liking, resistance to bruising during harvesting and transportation, and shelf-life. So far, most of the breeding studies applied to blueberry are focused on mechanical attributes, while the relevance of acoustic and other mouthfeel properties remains elusive at this point. Studies assessing mechanical texture indicated that in blueberry multiple components are contributing to texture [16]. To date, no study has been conducted in blueberry to evaluate genetic parameters associated to texture attributes. To fill this gap, here we used TA to evaluate a large diversity of texture attributes in SHB and NHB populations. Our results indicate that both firmness (also known as Chord stiffness), usually measured with the compression method with FirmTech equipment, and SFM, measured here with the TA, represent the slope of the chord drawn between any specific point on the force–deformation curve. Slight deviations from these measurements obtained with the two machines are expected since the points set by the operators are specific to each experiment and can affect the slope values [6]. Mean SFM value in SHB was higher than NHB. This result is consistent with the blueberry literature, indicating that SHB are overall firmer than NHB genotypes [17]. However to note, that SFM is not one of the parameters that best explains the difference among genotypes in the NHB and SHB populations (Fig. 1) and has not been identified as a key trait to explain sensorial texture or postharvest texture changes [16]. This highlights the importance of using methods that are more aligned with the texture measurements by the TA.

At the breeding level, molecular breeding tools could leverage early selections and increase genetic gains. Two main forms of marker-assisted breeding have been successfully applied in fruit crops: (i) genomic selection (GS), that relies on all markers considered simultaneously to predict the genetic merit of an individual for more complex traits; (ii) marker-assisted selection (MAS), that focuses on the use of previously identified targeted molecular markers for more oligogenic traits. Nonetheless, both methods can be integrated in the breeding pipeline, with MAS used in early-stage selection, for example, to screen a large number of seedlings for simple traits, followed by a GS approach to predict the genetic merit for complex traits for a reduced number of individuals out of the MAS selection.

Prior to the predictive and association analyses, we took advantage of this large and diverse blueberry collection, including two representative genetic pools, to investigate some population genetic parameters. First, we observed a rapid LD decay and a large frequency of pairwise markers in low LD, in both populations. This result is fairly expected given the cross-pollination nature of the species and it justifies the use of high-density marker for all the downstream genetic analyses. Also relevant, we shed some light on sweeps across the genome, which opens an important window of opportunities to explore signature of selection. In particular, at the end of the chromosome 5, we noticed large differences in the allele frequencies across both populations (large Fst), that could be better explored in additional studies.

In terms of genetic architecture, in this study, we identified texture traits to have a complex genetic architecture, with low to moderate genomic heritability values and marker associations explaining only a small fraction of the genetic variation through GWAS analyses. Therefore, a GS breeding approach was explored for texture traits. Predictive abilities were larger when models were calibrated and tested within NHB and SHB populations separately. In contrast, and as expected, poor accuracies were obtained for inter-population predictions, hindering the use of the same predictive model across breeding programs for these two types of blueberries.

To define potential targets for MAS, on the other hand, we relied on GWAS analyses and gene mining to narrow down potential candidates related to three important traits (YM20_BuStr, F1mm and DFM). In a total of 17 texture traits measured via TA, we have focused our discussions on traits that have shown previous evidence of being associated with sensory and post-harvest characteristics [8, 11, 16]. For example, when exploring the shelf-life predictability in blueberry [16] showed that DFM is the best predictor for overall change in texture. In the same studies, the authors reported that F1mm trait showed a good ability to separate firm and soft genotypes, suggesting that F1mm has a close connection with firmness sensory perception. The use of Y20_BuSt, on the other hand, was pointed out in our PCA results as one of the most important attributes to differentiate SHB and NHB blueberries, and for this reason, was further investigated.

The genomic regions surrounding significant associations for two of the most relevant traits (YM20_BuStr and DFM) showed promising candidate genes that could be potential targets for future functional validation. At the molecular level, textural changes during ripening and postharvest of fleshy fruits have been strongly attributed to cell wall modifications [18]. For YM20_BuStr, we found candidate genes encoding enzymes that could directly modify plant cell wall and have been shown to contribute to fruit softening in several species, such as: a “*xyloglucan endotransglucosylase/hydrolase (XTH)*” that plays a role in disassembling xyloglucans and loosening the cell wall [19, 20]; a “*pectate lyase (PEL)*”, which degrades de-esterified pectin in the primary wall and middle lamella [21, 22]; and a “*pectinesterase/pectinesterase inhibitor (PME)*”, which acts in the modification of cell walls via demethylesterification of pectins [23].

In addition to cell wall modifying enzymes, genes encoding transcription factors that could regulate cell wall targets were also identified as candidate genes. For YM20_BuStr, we found transcription factors from different families whose members have been reported to be involved in fruit softening, such as “*NAC domain-containing protein (NAC)*” [24] a “*squamosa promoter-binding-like protein (SPL)*” [25], an “*ethylene-responsive transcription factor-like protein (ERF)*” [18], a “*BES1 BZR1 homolog protein*” [26], and a “*C2H2 zinc finger protein*” [27].

Genes that are part of broader regulatory and hormonal mechanisms of fruit ripening were also detected. For DFM, we found a gene encoding a “*12-oxophytodienoate reductase 3 (OPR3)*”, which is involved in the biosynthesis of jasmonic acid and oxylipins. Jasmonate is a plant hormone involved in fruit ripening, and its external application has been shown to affect texture in different fruit species by regulating cell wall enzyme activity [28, 29]. This gene was also found to be upregulated in a melon line with higher flesh firmness and whole fruit hardness, but lower juiciness, when compared to its parental genotype [30]. For YM20_BuStr, genes encoding a “*flavin-containing monooxygenase (FMO)*” were found in different peaks. A member of this family, named YUCCA, catalyzes a rate-limiting step in the tryptophan-dependent auxin biosynthesis [31]. In peaches, an insertion of a transposon-like sequence in the 5′-flanking region of the *YUCCA* gene was found to cause the stony hard phenotype, likely due to a reduction of the auxin levels at the late-ripening stage, resulting in low ethylene production and inhibition of fruit softening [32].

Among other categories of candidate genes highlighted, there was a “*histone deacetylase 15 (HDT)*” that could be recruited to epigenetically repress key ripening genes [33]. In tomato, a histone deacetylase (SlHDT3) was found to regulate fruit softening, altering the expression of ethylene biosynthesis genes and cell wall metabolism [34]. We also pinpointed genes encoding “*wall-associated receptor kinase (WAK)*” that participate in cell integrity and are receptors for pectin and oligogalacturonic acids binding in the cell wall. In grapes, WAKs genes (*VvWARK2* and *VvWARK8*) were found in a stable QTL for mesocarp firmness pericarp puncture hardness [35] and were highly expressed in a firm-fleshed compared to a soft-fleshed cultivar [36]. A similar pattern of expression of WAK genes was found when comparing normal and mealy peach fruits in cold storage [37].

For future directions, we first highlight the representativeness of this research. Genetic parameters of 20 texture traits in blueberry were estimated, using two representative breeding populations of NHB and SHB. Multiple candidate genes were pinpointed, and genomic selection reveals a valid alternative to predict texture. Despite important results, a measure of caution should be taken in the interpretation of the genetic parameters. Data collection was performed in a single year and was potentially upwardly biased by Genotype-by-Environment interactions. However, an experiment of this magnitude cannot be easily repeated. Another important aspect is the real impact of the TA values on consumer preferences. Some preliminary studies in fruits have shown a correlation between some TA metrics with crunchiness and other textural aspects [5]. However, the main texture attributes measured via TA analyses associated to consumer acceptability are still elusive and should be further explored in future sensory analyses.

Altogether, our contributions in this paper are threefold in that (i) we showed that texture traits can differentiate genotypes from NHB and SHB groups; (ii) we computed genetic parameters and suggested that most of the texture attributes have a complex genetic architecture; (iii) we draw attention to the potential of genomic selection to accelerate and increase genetic gains for these traits; and (iv) we provided GWAS hits and candidate genes to be further validated.

## Material and methods

### Plant material

In this study, we used two breeding populations from NHB and SHB blueberry types. These breeding programs are conducted independently at USDA-ARS and Oregon State University (Corvallis, OR) and at University of Florida (Gainesville, FL), respectively. Both programs use the basic crossing design following a phenotypic recurrent selection model where parents are selected to maximize the genetic gains and minimize potential inbreeding [14].

The NHB population used in this study includes a total of 1065 genotypes. The population was further divided into genetic (GEN) and validation (VAL) study sets. The GEN study set comprises 952 individuals in the first stage of evaluation in the breeding program, derived from 85 controlled crosses made from 51 different parents. The VAL study set includes 113 individuals of advanced selections and cultivars from the NHB breeding program. Both the VAL and GEN study sets were planted in 2016 and 2017 in a high-density nursery at the Oregon State University Lewis Brown Farm in Corvallis, OR. For the NHB population, phenotypic information was collected in 2020, 2021, and 2022.

The SHB population used in this study comprises a total of 992 genotypes. Similarly to NHB, we divided the SHB population into two sets: genetic (GEN) and validation (VAL). The GEN set comprises 879 diverse individuals evaluated in the first stages of the breeding program, derived from 160 controlled crosses made from 205 selected parents in February of 2017 and 2018. The GEN population was established as a high-density nursery at the Plant Science Research and Education Unit (PSREU) in Citra, FL. The VAL set consists of 113 genotypes, including advanced selections and cultivars planted under commercial field practices at Waldo, FL. For the SHB population, phenotypic information was collected across two harvest-years (2021 and 2022).

### Texture analyses

For each genotype from both NHB and SHB breeding programs, 10 berries that were fully ripe and free of visible external defects/disease were selected. Texture was evaluated using a penetration method implemented in a TA.XTPlus Texture Analyzer (Stable Micro Systems, Hamilton, MA, USA) as previously described by Giongo *et al.* [11]. The 10 berries were placed into a plastic condiment cup, held for 24 h at 4°C prior to measurement and warmed to room temperature prior to analysis. The penetration test used a 2 mm flat cylinder probe (TA-52) and test speed of 1 mm s^−1^ to a final depth of 90% strain onto the equatorial axis of the fruit, perpendicular to the probe. The trigger force was set to 0.5 N with 100 data points collected per second. Using this method for each berry, 17 mechanical parameters were derived from the force deformation curve: distance to maximum force (mm, DFM), slope maximum force (N mm^−1^, SFM), area maximum force (N mm, AFM), linear distance to maximum force (LDFM), area internal force (N mm, AIF), number of internal peaks (#, NIP), force linear distance (FLD), area force linear distance (N mm, AFLD), peak force at 1 mm (N, F1mm), maximum force (N, FM), mean internal force (N, MIF), and five Apparent Young’s modulus (YM) (see Table 1 for detailed description). Data were extracted, and berries with measurements of F1mm <0.05 (minimum force detection threshold) were removed.

We also compared results from the TA.XTPlus Texture Analyzer with traditional deflection results measured using the FruitFirm 1000 equipment (CVM Inc., Pleasanton, CA, USA). To this end, we designed a straightforward experiment where a pool of 100 random samples was measured in the same order in both machines. For the FruitFirm device, a load cell is used to determine a force–deformation value as a measure of firmness. Compression force (g/mm) from the FruitFirm 1000 was recorded and correlated with the texture attributes described in Table 1.

### Genotypic data

Young leaves from each genotype were collected and submitted for DNA extraction, library preparation, and targeted genotyping by sequencing at RAPiD Genomics (LGC Group, Gainesville, FL). A probe panel comprising 22 000 loci were used for targeted genotyping using the Flex-Seq® Ex-L platform (Flex-Seq Panel Code: FS_1903) as described by [12]. After library preparation, samples were paired-end sequenced using 150 cycles at Illumina NovaSeq platform. Raw reads were cleaned and trimmed, and the remaining reads aligned against the “W85 P0” reference genome [38] using Mosaik v.2.2.3 [39]. SNPs were called for all NHB and SHB genotypes at once with FreeBayes v.1.3.2 using the probe intervals as targets [40]. A total of 173 256 SNPs were identified. Loci were then filtered out by applying the following criteria: only biallelic locus; no monomorphic; maximum missing data of 50%; and minimum and maximum filtering depth of 15 and 150 reads, respectively. A total of 124 070 SNPs were kept after these filtering steps. Read counts were then extracted per allele per individual using vcftools v.0.1.16 [41] and subsequently used to probabilistically assign genotypic classes using the “norm model” in the updog R package v.2.1.0 [42]. From the sequence of this work, we divided our analysis in three data sets contain individuals from the SHB population, NHB population, and grouping both populations. For all the three data sets, we filtered out SNPs with minimum allele frequency less than 1%. The final number of molecular markers retained in the SHB, NHB, and both populations simultaneously were 101 386; 103 187; and 113 421 SNPs, respectively. The genotypic scores were further used in GWAS and GS analyses.

### Signature of selection and population structure

Pearson correlation tests (r2) were performed for pairwise LD estimation per chromosome, as described by de Bem Oliveira [43]. Signature of selections across the chromosomes were investigated by quantifying allelic frequency differences between SHB and NHB populations using the Wright’s Fst statistics [44]. To assess the genetic diversity, the PCA was performed using the marker-based relationship matrix as input. Tetraploid genomic relationship matrices were computed with the AGHmatrix R-package [45]. Further, we investigated the genetic structure in each population using the probabilistic model implemented in the STRUCTURE software [46], for a range of K = 2 to 10. The burn-in period length was set to 20 000 and a total of 200 000 Markov Chain Monte Carlo (MCMC) chains. Because the STRUCTURE algorithm is sensible to the final number of markers included in the analyses, we considered a SNP pruning method to select equidistant markers across all the chromosomes. Thus, a total of 5166 and 4882 SNPs were selected for SHB and NHB, respectively. The best fitting K was identified with STRUCTURE HARVESTER website [47].

### Phenotypic analyses and genetic analyses

Descriptive analyses were performed in both breeding populations to check general trends and phenotypic variability. Differences between trait performances of SHB and NHB populations were quantified via analysis of variance (ANOVA) using the *lm()* function in the R software. Additionally, PCA was carried out to summarize and visualize the variability in both populations, using the FactoMineR R package [48]. To complement the multivariate analyses, we relied on an unsupervised statistical learning method to quantify the importance of texture traits on separating SHB and NHB genotypes. To this end, a Random Forest model was implemented using the default options from the randomForest R package [49] with the number of trees to grow equal to 500 and number of variables randomly sampled equal to p/3, where p is the number of variables.

For the phenotypic model, as multiple fruits were collected from the same individual, we estimated the so-called permanent environment effect and computed repeatability values using the following univariate linear mixed model: $\mathbf{y= X\beta + Zu+ Wp +e}$, where, **y** is the vector of phenotypic data, $\beta$ is the vector of year effect; **u** is a vector of random additive effects following a normal distribution with zero mean and (co)variance matrix $A{\sigma}_u^2$, where **A** is the additive relationship matrix and ${\sigma}_a^2$ is the additive genetic variance; **p** is the vector of permanent environmental effect and non-additive genetic variance that is independently distributed with means of zero and variance ${\sigma}_{pe}^2$; and **e** is the vector of residual effect following a normal distribution with zero mean and (co)variance matrix ${\sigma}_e^2$. **X, Z**, and **W** are incidence matrices relating the fixed and random effects to measurements in vector **y**. Mixed models were implemented using the ASReml-R 4.0 software [50], while the **A** matrix was computed using the AGHmatrix software [45]. Repeatability was computed as ${r}^2=\frac{\sigma_u^2+{\sigma}_p^2}{\sigma_u^2+{\sigma}_p^2+{\sigma}_e^2}$, following a similar strategy reported in [51]. For all the subsequent genomic analyses, we estimated the empirical BLUEs associated to the genotypic effects to be used as the observed phenotypes.

### Genome-wide association analyses

The SNP-trait association analyses were based on a linear mixed model, accounting for population structure (**Q**) and the polygenic term (**K**) as implemented in the GWASpoly R package. The following model was fitted for each trait: $y= W\alpha + x\beta + Zu+e$, where *y* is an *n*-vector of observed phenotypes, pre-corrected for the environment effects for *n* individuals, **W** is an *n* x *c* incidence matrix of covariates (fixed effects); and $\alpha$ is a *c*-vector of the corresponding coefficients including the intercept; **x** is an *n*-vector of marker genotypes, encoded in the tetraploid version after estimated the allele dosages; $\beta$is the fixed effect of the marker; **Z** is the incidence matrix that maps the genotypes to observation; **u** is an *n*-vector of the random polygenic effects, with $u\sim MVN\left(0,G{\sigma}_u^2\right)$ where **G** is a known *n* × *n* genomic relationship matrix; and **e** is a random *n*-vector error term, with $e\sim MVN\left(0,I{\sigma}_e^2\right)$ where **I** is an *n* x *n* identity matrix. MVN denotes the n-dimensional multivariate distribution.

To correct for population structure, we used the five first principal components as covariates in the GWAS model and used the leave-one-chromosome-out (LOCO) approach to increase the statistical power by avoiding to double fitting of the candidate marker in the model — phenomenon commonly reported in the GWAS literature as “proximal contamination” [52]. Hypothesis tests were conducted for the additive gene action, and statistical significance defined using the “M.eff” method—a Bonferroni-type correction that accounts for an effective number of markers considering LD information. The relative importance of each marker was tested using a likelihood ratio test under a backward elimination as implemented in the “fit.QTL” function.

### Annotation of genetic associations

The genomic position of significant SNPs detected through GWAS analyses for the most relevant traits (YM20_BuStr, F1mm, and DFM) was used to search for candidate genes. Genes within 100 kb on each side of the SNPs were retrieved from the reference genome “W85 P0” and their functional in silico annotation performed using PANNZER2 [53] and eggNOG-mapper servers [54]. For significant SNPs located in an interval less than 1 Mb, the entire region in between was screened for candidate genes. Functional descriptions were further examined from the literature.

### Genomic prediction

A genomic best linear unbiased prediction (GBLUP) model was fitted to predict the genetic merits, as follow: $\mathbf{y={1}^{\prime}\mu + Zu+e}$**,** where **y** is an *n*-vector of phenotypic observations after corrected for the year effect, $\mu$is the population mean, and **1** is a vector of same dimension of y being all elements equal to 1, fixed effect; **Z** is the design matrix linking the phenotypic records to genotype random effect, with $u\sim MVN\left(0,G{\sigma}_u^2\right)$ where **G** is a known *n* x *n* genomic relationship matrix built using the AGHmatrix R package [45]; and $e\sim MVN\left(0,I{\sigma}_e^2\right)$ where **I** is an *n* x *n* identity matrix. Variance components estimated from this model were used to compute a narrow-sense “genomic-based heritability”, as: $E\left[{h}^2\right]\approx \frac{\sigma_u^2\left(1+f\right)}{\sigma_u^2\left(1+f\right)+{\sigma}_e^2}$, where $\left(1+f\right)$ is the mean of the diagonal elements of the **G** matrix, that is equivalent to the inbreeding coefficient (*f*) of the current population. Mixed models were implemented using the ASReml-R 4.0 software [50].

Model performance was evaluated using inter and intra-population prediction models. For intra-population analyses, predictive abilities were computed as the Person’s correlation between predicted and observed values within each population (NHB and SHB) and using a 10-fold cross-validation scheme. For inter-prediction analyses, we tested the predictive performance across breeding populations. Namely, models were trained in the SHB population and used to predict individuals in the NHB population and vice versa.

## Supplementary Material

Web_Material_uhae233

## Data Availability

The genomic and phenotypic data are available as supplemental material.
