# Supplementary material for: Inference of the genetic basis of fruit texture in highbush blueberries using genome-wide association analyses
Source: Hortic Res. 2024 Aug 21;11(10):uhae233. doi: 10.1093/hr/uhae233 (PMC11489598; doi:10.1093/hr/uhae233)

| **trait** | **average values** | | **anova** | | |
| --- | --- | --- | --- | --- | --- |
|  | **meanSHB** | **meanNHB** | **SHB vs NHB** | **SHB (GEN vs. VAL)** | **NHB (GEN vs. VAL)** |
| FIRM_n | 0.049676373 | -0.122183338 | TRUE | FALSE | FALSE |
| SKIN_n | -0.00545807 | -0.400940463 | TRUE | TRUE | FALSE |
| ELAST | 0.003623224 | -0.134256054 | TRUE | TRUE | TRUE |
| FIRM_SKIN | -0.030579244 | -0.187429327 | TRUE | FALSE | FALSE |
| SKIN_a | 0.074164917 | -0.59667371 | TRUE | TRUE | TRUE |
| LDIST | 0.016283012 | -0.318818077 | TRUE | TRUE | TRUE |
| FIRM_int | -0.007848948 | -0.369804524 | TRUE | TRUE | TRUE |
| FIRM_a | 0.100121561 | -0.271037647 | TRUE | FALSE | TRUE |
| GRAIN | -0.148107773 | -0.140731508 | FALSE | FALSE | TRUE |
| LDFORCE | 1.378202624 | 8.207180554 | TRUE | FALSE | TRUE |
| AREA | 0.191716812 | -0.589862996 | TRUE | TRUE | FALSE |
| YM10PD_Cond | 29.14695405 | 263.0458268 | TRUE | FALSE | TRUE |
| BuStr | -0.088370287 | -0.492724149 | TRUE | TRUE | TRUE |
| YM20_BuStr | 5.483628753 | 66.86565822 | TRUE | FALSE | TRUE |
| YM100_BuStr | 6.694128232 | 27.69002663 | TRUE | FALSE | TRUE |
| YM80_BuStr | 7.091732641 | 65.5051832 | TRUE | FALSE | TRUE |
| YM1.2 | 12.97374431 | 211.6238543 | TRUE | TRUE | TRUE |

**Table S1:** Comparison between texture-related traits computed in Southern and Northene Highbush Blueberries (SHB and NHB). After standardized the values for both populations, we reported the average values per population. For ANOVA, we tested a model comparing differences across both populations (SHB vs. NHB) and within population, we compared the average values reported for the GEN and VAL data set. Results reported as TRUE indicated statistical significance differences at the global level of 0.05.

**Figure S1** Repeatability analyses for 14 texture -related. For the phenotypic model, as multiple fruits were collected from the same individual, we estimated the so-called permanent environment effect and computed repeatability values (non-additive effect) using mixed model. The y-axis is representing the variance projection. The variance component associated with the additive effect was estimated using pedigree information.


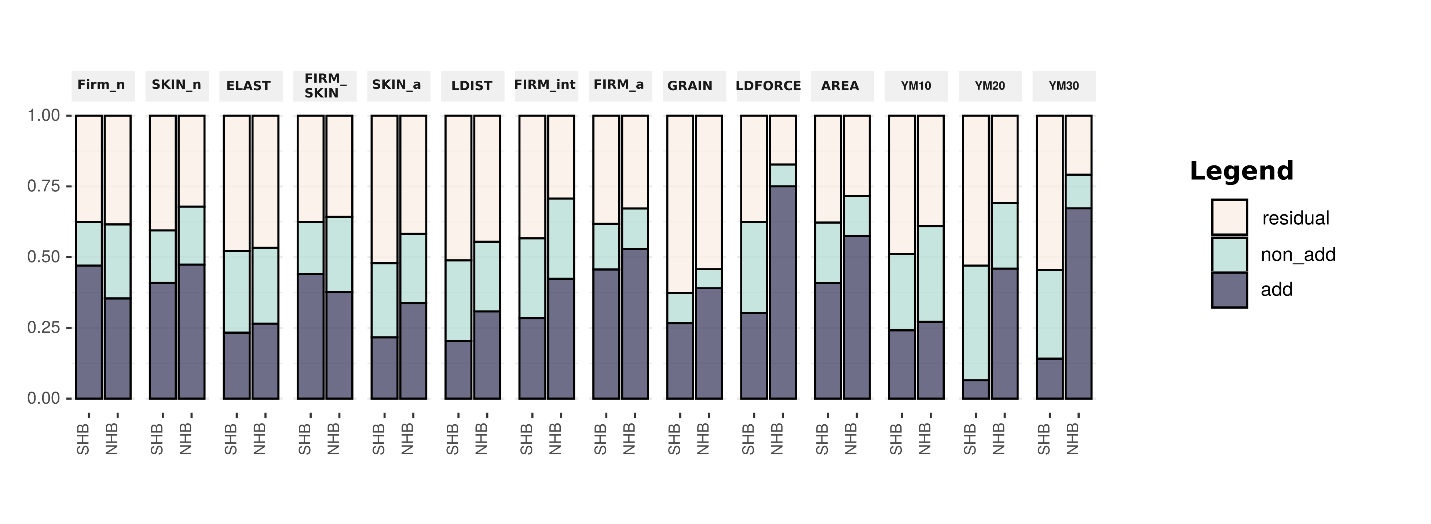


**Figure S2.** Manhattan plots for texture traits evaluated in the Florida (FL) Southern Highbush Blueberry (SHB). The traits under evaluation are FIRM_n (F1mm), SKIN_n (FM), ELAST (DFM), FIRM_SKIN (SFM), SKIN_a (AFM), LDIST (LDFM), FIRM_int (MIF), FIRM_a (AIF), GRAIN (NIP), LDFORCE (FLD), AREA (AFLD) and the Young modules (YG).


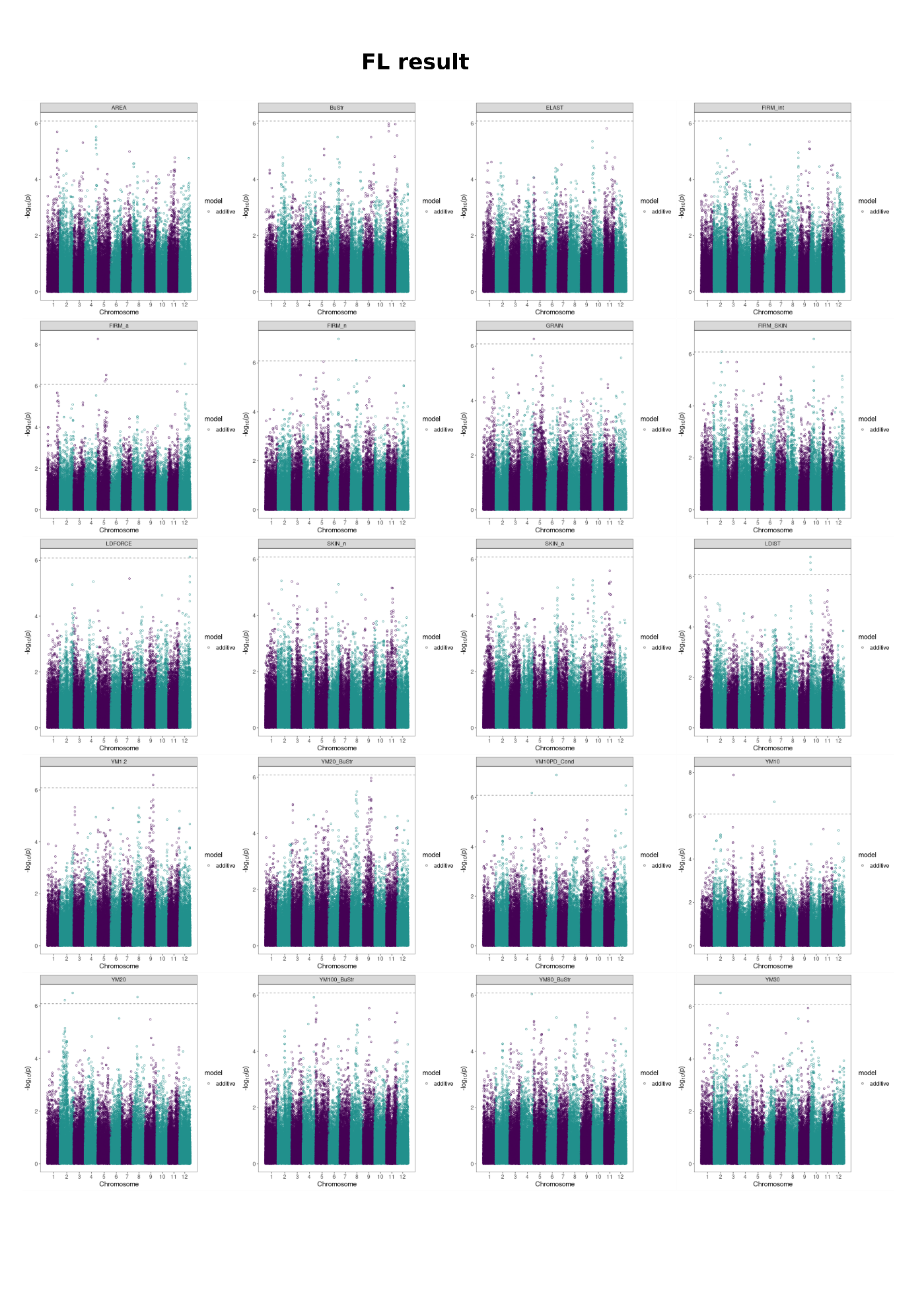


**Figure S3.** Manhattan plots for texture traits evaluated in the Oregon (OR) Northen Highbush Blueberry (NHB). The traits under evaluation are FIRM_n (F1mm), SKIN_n (FM), ELAST (DFM), FIRM_SKIN (SFM), SKIN_a (AFM), LDIST (LDFM), FIRM_int (MIF), FIRM_a (AIF), GRAIN (NIP), LDFORCE (FLD), AREA (AFLD) and the Young modules (YG).


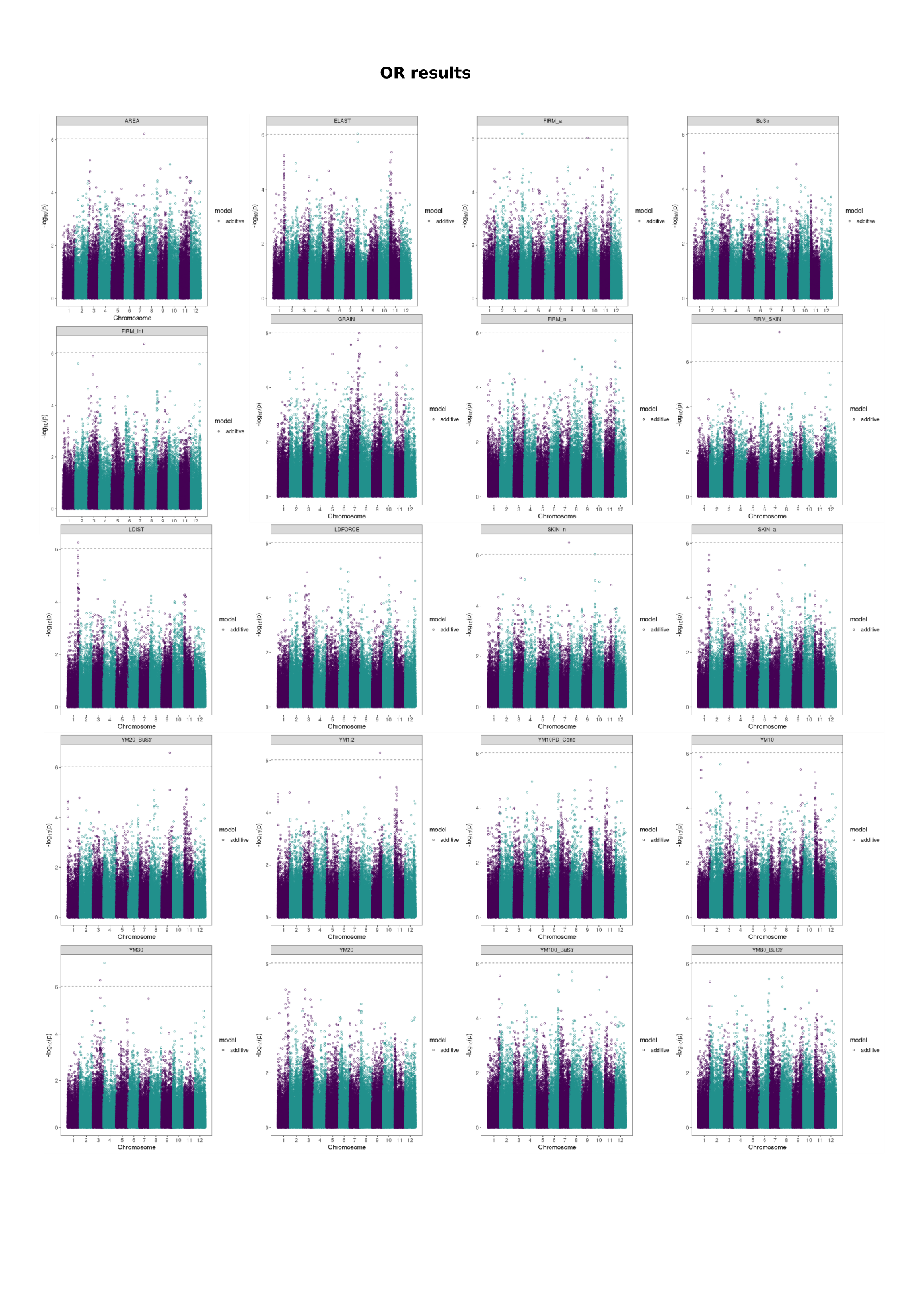


**Figure S4.** Manhattan plots for texture traits evaluated combining Florida (FL) Southern Highbush Blueberry (SHB) and Oregon (OR) Northen Highbush Blueberry (NHB). . The traits under evaluation are FIRM_n (F1mm), SKIN_n (FM), ELAST (DFM), FIRM_SKIN (SFM), SKIN_a (AFM), LDIST (LDFM), FIRM_int (MIF), FIRM_a (AIF), GRAIN (NIP), LDFORCE (FLD), AREA (AFLD) and the Young modules (YG).


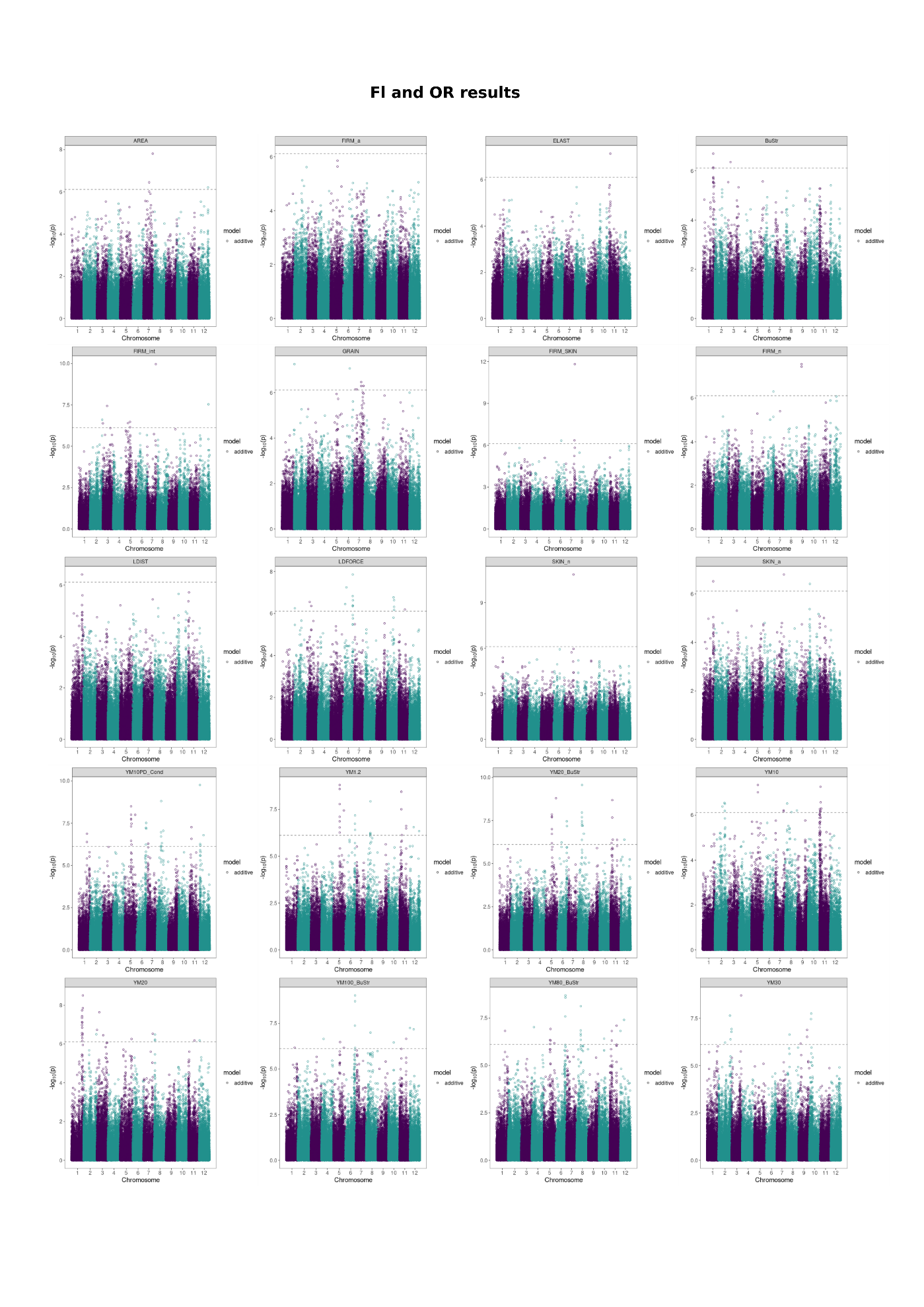

Supplement: Web_Material_uhae233 [file web_material_uhae233.zip › SM1.docx]
